# Predicting hypoglycemia after treatment of hyperkalemia with insulin and glucose (Glu-K60 score)

**DOI:** 10.1186/s12873-022-00748-9

**Published:** 2022-11-12

**Authors:** Weerapriya Kijprasert, Nilanut Tarudeeyathaworn, Chananthita Loketkrawee, Thidarat Pimpaporn, Pornpiyapat Pattarasettaseranee, Theerapon Tangsuwanaruk

**Affiliations:** 1 Faculty of Medicine, Chiang Mai University, Chiang Mai, Thailand; 2 Department of Emergency Medicine, Faculty of Medicine, Chiang Mai University, 110 Inthawaroros Road, Sribhumi, Amphoe Muang, Chiang Mai, 50200 Thailand

**Keywords:** Hyperkalemia, Hypoglycemia, Insulin, Prediction

## Abstract

**Background:**

Hyperkalemia can lead to fatal cardiac arrhythmias. Ten units of intravenous (IV) regular insulin with 25 g of glucose is the mainstay for treating hyperkalemia. However, the most important complication of this treatment is hypoglycemia. We aimed to develop a scoring model to predict hypoglycemia after the treatment of hyperkalemia.

**Methods:**

A retrospective study was conducted at a university-based hospital between January 2013 and June 2021. We included the hyperkalemic patients (> 5.3 mmol/L) who were ≥ 18 years old and treated with 10 units of IV regular insulin with 25 g of glucose. Incomplete data on posttreatment blood glucose, pregnancy, and diabetes mellitus were excluded. Endpoint was posttreatment hypoglycemia (≤ 70 mg/dL or ≤ 3.9 mmol/L). Multivariable logistic regression was used to establish a full model and a subsequently reduced model using the backward elimination method. We demonstrated the model performance using the area under the receiver operating characteristic curve (AuROC), calibration plot, and Hosmer–Lemeshow goodness-of-fit test. Internal validation was done with a bootstrap sampling procedure with 1000 replicates. Model optimism was estimated.

**Results:**

Three hundred and eighty-five patients were included, with 97 posttreatment hypoglycemia (25.2%). The predictive model comprised the following three criteria: age > 60 years old, pretreatment blood glucose ≤ 100 mg/dL (≤ 5.6 mmol/L), and pretreatment potassium > 6 mmol/L. The AuROC of this model was 0.671 (95% confidence interval [CI] 0.608 to 0.735). The calibration plot demonstrated consistency with the original data. Hosmer–Lemeshow goodness-of-fit test showed no evidence of lack-of-fit (*p* 0.792); therefore, the model was also fit to the original data. Internal validation via bootstrap sampling showed a consistent AuROC of 0.670 (95% CI 0.660 to 0.670) with minimal model optimism. A high risk for posttreatment hypoglycemia was indicated if the patient met at least one of those criteria. Sensitivity and specificity were 95.9% and 14.9%, respectively.

**Conclusion:**

High risk was indicated when at least one of the criteria was met: age > 60 years old, pretreatment blood glucose ≤ 100 mg/dL (≤ 5.6 mmol/L), and pretreatment potassium > 6 mmol/L. Blood glucose levels should frequently check in the high-risk group.

**Trial registration:**

TCTR20210225002 (www.thaiclinicaltrials.org).

## Introduction

Hyperkalemia is one of the critical conditions causing sudden death [1–3]. Among elderly patients, hyperkalemia was present in 2.6% of Emergency Department (ED) visits and 3.5% of hospital admissions [4]. Hyperkalemia is characterized by elevated serum potassium concentration (greater than 5.3 mmol per liter [mmol/L]) [5]. Hyperkalemia leads to many symptoms (diarrhea, vomiting) and clinical signs (depressed tendon reflex, paralysis, paresthesia, or even cardiac arrhythmia, a life-threatening condition) [5]. Several treatment options for hyperkalemia, include stabilization of the cardiac membrane, shifting extracellular potassium into cells, and increasing the elimination of potassium from the body. In a hyperkalemic emergency, intravenous insulin accompanied by glucose is helpful for management because of its rapid onset and effective hypokalemic effect [6]. Seventy percent of potassium is stored in skeletal muscles. Insulin promotes Na^+^-H^+^ antiporters (sodium/hydrogen exchanger-1 [NHE1]), leading to sodium influx into the muscle cells, then increased concentration of intracellular sodium stimulates the sodium–potassium-adenosine triphosphatase (Na^+^-K^+^ ATPase) transporter on the cell membranes. Also, insulin promotes Na^+^-K^+^ ATPase translocation from intracellular storage to the cell membrane. Eventually, extracellular potassium shifts into the cell which exchanges with intracellular sodium and decreases serum potassium [6–10].

Hypoglycemia is a common complication of insulin treatment in hyperkalemic patients [6, 9]. To prevent posttreatment hypoglycemia, glucose is recommended to be given with insulin among patients without hyperglycemia (blood glucose < 250 mg per deciliter [mg/dL] or < 13.9 mmol/L) [8]. The current regimen of hyperkalemic patients is managed by 10 units of regular insulin with 25 g of glucose intravenously (IV) [6, 9]. Hypoglycemia after treatment of hyperkalemia ranges from 8.7% to 75% [6, 9, 11–13]. Hypoglycemia is defined as a blood glucose level of 70 mg/dL (3.9 mmol/L) or less. Hypoglycemia can have crucial effects on patients, not only medical complications that cause patients at risk of serious harm such as neurologic symptoms such as dizziness and convulsions. Also, hypoglycemia can cause psychological symptoms such as anxiety or even coma and death. Moreover, hypoglycemia can give rise to non-medical complications, which are the causes of a patient's stress, such as increased healthcare costs and length of stay [14]. However, there was no available predictive score to predict hypoglycemia after treating hyperkalemia with insulin and glucose.

This study aimed to develop a predictive scoring model that can predict the occurrence of hypoglycemia after administering 10 units of IV regular insulin with 25 g of glucose for the treatment of hyperkalemia.

## Methods

### Study design and settings

The study was a retrospective observational study with a predictive score development, including internal validation. The data of patients with hyperkalemia in the ED, the outpatient department (OPD), and the inpatient department (IPD) in Maharaj Nakorn Chiang Mai Hospital, tertiary care, university-based hospital between January 2013 and June 2021 were collected from the electronic medical records. The study was conducted according to the guidelines of the Declaration of Helsinki, and the protocol was approved by the Research Ethics Committee of the Faculty of Medicine, Chiang Mai University (protocol code 022/2021 and date of approval: 20/01/2021). This study was prospectively registered in the Thai Clinical Trials Registry (TCTR20210225002) on 25/02/2021. The Research Ethics Committee of the Faculty of Medicine, Chiang Mai University waived the need for informed consent due to the retrospective design. We followed the Transparent Reporting of a multivariable prediction model for Individual Prognosis or Diagnosis (TRIPOD) Statement.

### Selection of patients

Eligible criteria were patients who were 18 years old or more, diagnosed with hyperkalemia (serum potassium > 5.3 mmol/L), and treated with 10 units of IV regular insulin and 25 g of glucose. We excluded patients without data on posttreatment blood glucose (our endpoint), pregnancy, and diabetes. We excluded pregnant patients because decreased insulin sensitivity leads to hyperglycemia and might be gestational diabetes mellitus [15]. We excluded diabetic patients because a previous study reported that non-diabetic patients had an increased risk of posttreatment hypoglycemia [11, 16].

### Data collection

Patient data in the hospital’s electronic medical records were retrospectively reviewed by researchers using the tenth revision of the International Statistical Classification of Diseases and Related Health Problems (ICD-10) code as hyperkalemia code (E87.5). We collected the data using a secure web-based data collection system through the Research Electronic Data Capture (REDCap) platform [17].

### Study variables and candidate predictors

Baseline characteristics were recorded including: age, sex, weight, height, body mass index (BMI), comorbidity, body temperature, respiratory rate, heart rate, blood pressure, oxygen saturation, pretreatment blood glucose, pretreatment serum potassium, pretreatment serum creatinine, posttreatment blood glucose, posttreatment serum potassium, complete blood count, other blood chemistry, liver function test, final diagnosis, treatment location, and in-hospital mortality. The selection of candidate predictors was based on clinically relevant and previous evidence (see Sect. Handling of continuous predictors).

### Clinical endpoints

According to a previous study, the effect of intravenous regular insulin and glucose lasted within 6 h [7]. However, another study found that more than two-thirds of hypoglycemia occurred within 12 h [18]. Therefore, the lowest posttreatment blood glucose level within 12 h after the first insulin administration was planned and collected in this study. If the patient had the repeated dose of insulin or glucose (during the period of assessment the endpoint as the posttreatment hypoglycemia), only the lowest blood glucose before being given the repeated dose would be determined as the posttreatment blood glucose level. Due to the retrospective design, the frequency of the blood glucose testing was based on each attending physician's decision. The patients with posttreatment blood glucose ≤ 70 mg/dL (≤ 3.9 mmol/L) were defined as the posttreatment hypoglycemic group [19].

### Statistical methods

#### Fundamental statistical analysis

Baseline characteristics were presented as number, percentage, mean, standard deviation, median, and interquartile range (IQR) as appropriate. Data visualization was used to explore the normal distribution of continuous variables. Categorical variables were evaluated using Fisher’s exact test. The comparison of continuous variables among two groups was analyzed with a *t*-test or Mann–Whitney U test as appropriate.

The Stata version 16 (Stata Corp LLC, College Station, TX, USA) was used to calculate our predictors and all statistical analyses. Statistical significance was determined at two-sided *p* < 0.05.

### Model development

#### Management of missing data

The missing data were handled with multiple imputation methods before multivariable logistic regression. From our expert consensus, weight, height, and BMI might not be measured in some patients due to unawareness, local protocol in each department, or limited personnel resources in our setting. For missing data mechanisms, missing data due to unawareness of healthcare personnel was a missing completely at random (MCAR), and missing data due to differences in the local protocol in each department or limited personnel resources in our setting was missing at random (MAR). In general, the complete case analysis or ignoring missing data gives unbiased in MCAR but increases the standard error of the sample estimates owing to the reduced sample size [20]. In the case of MAR, the imputation method can give valid estimations of the study associations [21]. To decrease the standard error of the sample estimates and handle missing data mechanisms in the same variables of both MCAR and MAR, we planned to use an imputation method in handling missing data on weight, height, and BMI. We prespecified the procedure to handle this issue. If weight, height, or BMI were unavailable during that hyperkalemic visit, we used the most recent of previous data within 3 months. Then, if unfortunately those data were unavailable we planned to impute them with multiple imputation methods.

#### Handling of continuous predictors

The selection of candidate predictors was based on clinically relevant and previous evidence. Therefore, we preselected the following six candidate predictors: age, sex, BMI, pretreatment blood glucose, pretreatment serum potassium, and pretreatment serum creatinine. We categorized our continuous predictors into dichotomous data, including age, BMI, pretreatment blood glucose, pretreatment serum potassium, and pretreatment serum creatinine.

For the age of patients, a previous study reported that the age of 60 years or more represented a beginning of changes in glucose metabolism [22]. We planned to use a cut-off point greater than 60 years old.

In an aspect of sex, estrogen in females improves insulin sensitivity, resulting in more risk in females of developing hypoglycemia. A previous study revealed that females had an increased risk of hypoglycemia [18].

Lower BMI may cause increased insulin sensitivity, described as a decrease in free fatty acid mobilization. Moreover, those patients have less availability of hepatic glycogen storage, decreased glucagon and epinephrine secretion, and poor glycogenolysis and gluconeogenesis [23]. Therefore, they have the potential for hypoglycemia. We used a cut-off point of BMI below 18.5 kg/m^2^, considered underweight.

In the aspect of pretreatment blood glucose, a previous study revealed that pretreatment blood glucose between patients with and without posttreatment hypoglycemia was 104 mg/dL (5.8 mmol/L) and 162 mg/dL (9 mmol/L) (*p* 0.04) [16], respectively. Furthermore, the World Health Organization defined normal fasting blood glucose concentration ranging from 70 mg/dL (3.9 mmol/L) to 100 mg/dL (5.6 mmol/L) [24]. Therefore, we use a cut-off point of pretreatment blood glucose at 100 mg/dL (5.6 mmol/L) or less.

For the pretreatment serum potassium, a previous study discovered that pretreatment serum potassium was related to posttreatment hypoglycemia [12]. Another study reported potassium levels related to hypoglycemia were 6.7 ± 0.7 mmol/L [25]. To include the lowest potassium level related to hypoglycemia, we estimated 6.7 mmol/L minus the standard deviation of 0.7 mmol/L being 6 mmol/L. So, we divided the patients into two groups using a cut-off point greater than 6 mmol/L due to the minimal range of serum potassium associated with hypoglycemia being 6 mmol/L [25].

For serum creatinine, a prior study found that increased baseline serum creatinine was related to a high risk for hypoglycemia [26]. Another study reported that the lowest creatinine level related to hypoglycemia was 3.3 mg/dL (291.7 µmol/L) [25]. We divided the patients into two groups using a cut-off point greater than 3.3 mg/dL (291.7 µmol/L).

#### Multivariable modeling

All preselected clinically relevant candidate predictors (age, sex, BMI, pretreatment blood glucose, pretreatment serum potassium, and pretreatment serum creatinine) were included in the initially multivariable logistic regression model (full model). Then, the backward elimination method was performed to develop the reduced model with the least number of predictors with preserved predictive performance. We estimated the area under the receiver operating characteristic curve (AuROC) of the model to represent the model's predictive performance in each round. The non-statistically significant candidate predictor in the initial full model defined as *p* > 0.05 was gradually eliminated from the model. Then, we again estimated the *p*-value for each candidate predictor and the AuROC of the current multivariable logistic regression model. Similarly, the other non-statistically significant candidate predictor in that new current model (*p* > 0.05) was gradually eliminated. Those steps were repeated in multiple rounds fashion. In case of a significant decrease in the AuROC of each round's model, the eliminated candidate predictor in the previous step was re-added back into the model to preserve predictive performance. Another non-statistically significant candidate predictor in the current model (*p* > 0.05) was gradually eliminated from that current model. If each round had more than one non-statistically significant predictor, the predictor with the smallest magnitude of effect (defined as an odds ratio [OR] of nearly 1.00) was subsequently eliminated. The backward elimination steps were repeated until all the remaining candidate predictors in the model had *p* < 0.05, accompanied by the AuROC of the reduced model was well preserved. We used the last multivariable reduced model for the following risk score transformation step.

#### Simplified risk score transformation

After the multivariable modeling, we divided each predictor's regression coefficients (β coefficients) by the lowest of those coefficients in the final reduced model to transform into an integer number and use the integer number as the risk score (assigning the risk item score) for each predictor.

A parametric receiver operating characteristic curve (ROC) was used to demonstrate the discriminative performance of the model and presented as AuROC.

We planned to categorize the risk score as low-risk and high-risk patients and demonstrated the sensitivity, specificity, positive predictive value (PPV), negative predictive value (NPV), positive likelihood ratio (LHR +), negative likelihood ratio (LHR-), accuracy.

#### Sample size estimation

We based the sample size on the potential factor as female and the endpoint as posttreatment hypoglycemia (OR 0.4), including the incidence of females without hypoglycemia (40%) from a previous study [12]. Ninety-two events and 92 non-events of posttreatment hypoglycemia were required to provide at least 80% power with a two-sided significance level of 0.05. Therefore, we planned to include at least 200 patients with 92 events of posttreatment hypoglycemia.

#### Model performance and internal validation

We demonstrated the diagnostic model performance of the model in terms of discriminative ability (such as AuROC) and calibration. The model calibration, i.e., the agreement of the model prediction and observed event occurrence, was visualized via a calibration plot. The Hosmer–Lemeshow goodness-of-fit test was also estimated to detect the lack-of-fit of the model defined by the *p* < 0.05. Internal validation was done with a bootstrap sampling procedure with 1000 replicates. The model optimism was estimated and reported.

#### *Post-hoc analysis using posttreatment blood glucose* < *54 mg/dL (*< *3.0 mmol/L) as posttreatment level 2 hypoglycemia and level 3 hypoglycemia*

A joint position statement of the American Diabetes Association and the European Association for the study of diabetes proposed the following glucose levels when reporting hypoglycemia in clinical trials. Level 1 hypoglycemia was a glucose level of 54-69 mg/dL (3.0-3.8 mmol/L) as a glucose alert value. Level 2 hypoglycemia was a glucose level of less than 54 mg/dL (3.0 mmol/L) which indicated serious, clinically important hypoglycemia. Level 3 hypoglycemia was severe hypoglycemia regarding severe cognitive impairment requiring external assistance for recovery [19, 27]. Therefore, level 2 hypoglycemia (blood glucose level < 54 mg/dL or < 3.0 mmol/L) and level 3 hypoglycemia was also analyzed as posttreatment hypoglycemia.

## Results

### Patients

**Fig. 1:**
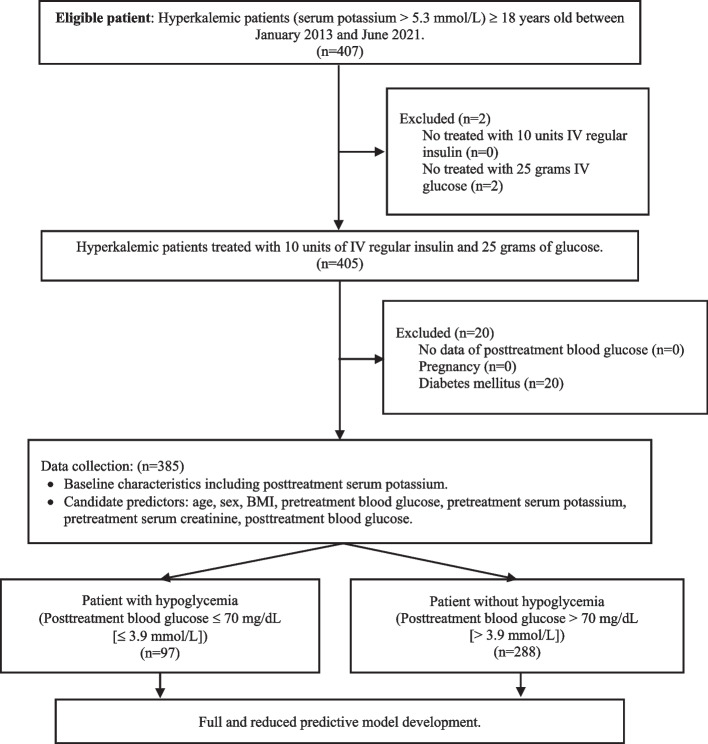
Study flow. Abbreviations: IV, intravenous; BMI, body mass index; mg/dL, milligram per deciliter; mmol/L, millimole per liter

A total of 385 hyperkalemic patients who were treated with 10 units of IV regular insulin and 25 g of glucose were included (Fig. 1). Baseline characteristics were similar except for age, comorbidity as CKD, pretreatment and posttreatment blood glucose, level of posttreatment hypoglycemia, white blood cell count, final diagnosis as hyponatremia, and treatment location in the hospital when diagnosed as hyperkalemia (Table 1). Posttreatment hypoglycemia was 97 patients (25.2%). Pretreatment serum creatinine demonstrated median 3.7 mg/dL (327.2 µmol/L), IQR 1.8 to 8.5 mg/dL (159.2 to 751.6 µmol/L) (minimum 0.3 mg/dL [26.5 µmol/L] and maximum 37.2 mg/dL [3287.5 µmol/L]). Among patients with posttreatment hypoglycemia, the median (IQR) of the duration of hypoglycemia was 253 min (190 to 435 min). Patients diagnosed with hyperkalemia at OPD had an increased risk of posttreatment hypoglycemia compared to IPD (OR 2.38, 95% confidence interval [CI] 1.18 to 4.8, *p* 0.015). However, no association was noted between the ED and IPD (OR 1.42, 95% CI 0.85 to 2.37, *p* 0.175). BMI was missing in 22.3%, pretreatment blood glucose in 4.4%, and pretreatment serum creatinine in 1.8% of the total population. Multiple imputation method was used for missing data.

**Table 1:** Baseline characteristics

Characteristics (*n* = 385)	Missing – n (%)	Overall	Posttreatment hypoglycemia (*n* = 97)	No posttreatment hypoglycemia (*n* = 288)	*P*-value*
Number		385	97 (25.2)	288 (74.8)	
Age – year†	0 (0)	59.1 ± 17.4	63 ± 16.8	57.7 ± 17.5	0.010
≤ 60 – n (%)∫		206 (53.5)	41 (42.3)	165 (57.3)	0.013
60 – n (%)∫		179 (46.5)	56 (57.7)	123 (42.7)	
Female – n (%)∫	0 (0)	149 (38.7)	37 (38.1)	112 (38.9)	1
Weight – kg†	76 (19.7)	56.3 ± 14.5	54.9 ± 12.6	56.8 ± 15.1	0.306
Height – centimeter†	86 (22.3)	160.5 ± 9.5	160.4 ± 9.7	160.5 ± 9.4	0.919
BMI – kg/m^2^†∫	86 (22.3)	21.7 ± 4.4	21.3 ± 3.8	21.9 ± 4.5	0.338
Below 18.5 – n (%)		64 (21.4)	20 (25.3)	44 (20)	0.529
18.5–22.9 – n (%)		123 (41.1)	30 (38)	93 (42.3)	
23.0–24.9 – n (%)		54 (18.1)	17 (21.5)	37 (16.8)	
25.0–29.9 – n (%)		48 (16.1)	11 (13.9)	37 (16.8)	
30.0 or more – n (%)		10 (3.3)	1 (1.3)	9 (4.1)	
Comorbidity – n (%)∫	0 (0)				
Hypertension		150 (39)	41 (42.3)	109 (37.9)	0.471
End stage kidney disease (ESKD)		114 (29.6)	32 (33)	82 (28.5)	0.441
Dyslipidemia		54 (14)	15 (15.5)	39 (13.5)	0.616
Chronic kidney disease (CKD)		46 (12)	18 (18.6)	28 (9.7)	0.029
Cirrhosis		32 (8.3)	10 (10.3)	22 (7.6)	0.401
Others		190 (49.4)	49 (50.5)	141 (49)	0.815
None		11 (2.9)	2 (2.1)	9 (3.1)	0.737
Body temperature – degree Celsius†	30 (7.8)	36.7 ± 1.0	36.8 ± 0.9	36.7 ± 1.0	0.564
Respiratory rate – per minute†	10 (2.6)	23 ± 6	23 ± 9	22 ± 7	0.403
Heart rate – per minute†	2 (0.5)	90 ± 22	88 ± 24	92 ± 21	0.197
Systolic blood pressure – mm Hg†	1 (0.3)	124 ± 32	126 ± 27	123 ± 33	0.405
Diastolic blood pressure – mm Hg†	1 (0.3)	73 ± 22	74 ± 21	73 ± 22	0.812
Oxygen saturation (SpO_2_) – %‡	40 (10.4)	98 (94, 100)	98 (92, 100)	98 (94, 100)	0.316
Pretreatment blood glucose – mg/dL (mmol/L)†	17 (4.4)	125 ± 51 (6.9 ± 2.8)	114 ± 59 (6.3 ± 3.3)	128 ± 48 (7.1 ± 2.7)	0.014
Pretreatment serum potassium – mmol/L†	0 (0)	6.4 ± 0.8	6.5 ± 0.6	6.4 ± 0.8	0.369
Pretreatment serum creatinine – mg/dL‡	7 (1.8)	3.7 (1.8, 8.5)	3.7 (2.3, 8)	3.7 (1.8, 8.6)	0.342
Posttreatment blood glucose – mg/dL (mmol/L)†	0 (0)	114 ± 63 (6.3 ± 3.5)	52 ± 13 (2.9 ± 0.7)	135 ± 59 (7.5 ± 3.3)	< 0.001
Level of posttreatment hypoglycemia – n (%)∫¶					
Level 1 hypoglycemia (54-69 mg/dL or 3.0-3.8 mmol/L)	0 (0)	45 (46.4)	45 (46.4)	0 (0)	< 0.001
Level 2 hypoglycemia (< 54 mg/dL or < 3.0 mmol/L)	0 (0)	35 (36.1)	35 (36.1)	0 (0)	
Level 3 (severe hypoglycemia)	0 (0)	17 (17.5)	17 (17.5)	0 (0)	
Posttreatment serum potassium – mmol/L†	16 (4.2)	5.3 ± 0.9	5.4 ± 0.8	5.3 ± 1	0.540
Complete blood count					
Hemoglobin – g/dL†	40 (10.4)	10 ± 2.7	9.7 ± 2.5	10.1 ± 2.7	0.230
Hematocrit – %†	10 (2.6)	31 ± 8	30 ± 8	31 ± 9	0.311
White blood cell count – 10^3^ cell/mm^3^‡	10 (2.6)	10.26 (6.9, 15.4)	8.55 (5.93, 14.66)	11.04 (7.26, 15.73)	0.030
Platelet count – 10^3^ cell/mm^3^‡	11 (2.9)	194 (135, 273)	183 (125, 242)	197.5 (138, 290)	0.176
Blood chemistry					
Blood urea nitrogen (BUN) – mg/dL‡	9 (2.3)	58 (38, 84)	53 (38, 81)	59 (39, 88)	0.660
Sodium – mmol/L†	1 (0.3)	134 ± 8	134 ± 8	134 ± 9	0.451
Chloride – mmol/L†	1 (0.3)	97 ± 10	98 ± 9	97 ± 10	0.162
Bicarbonate – mmol/L†	2 (0.5)	17 ± 6	17 ± 6	17 ± 6	0.972
Liver function test					
Total protein – g/dL†	66 (17.1)	6.7 ± 1.3	6.5 ± 1.3	6.7 ± 1.3	0.161
Albumin – g/dL†	61 (15.8)	3.2 ± 0.8	3.1 ± 0.8	3.2 ± 0.8	0.556
Globulin – g/dL†	67 (17.4)	3.5 ± 1	3.4 ± 0.9	3.6 ± 1	0.167
Alkaline phosphatase (ALP) – IU/L‡	71 (18.4)	112 (74, 200)	103 (70, 202)	113 (75, 196)	0.797
Cholesterol – mg/dL†	69 (17.9)	149 ± 68	146 ± 72	150 ± 67	0.647
Aspartate aminotransferase (AST) – IU/L‡	70 (18.2)	42 (24, 175)	42 (26, 208)	42 (23, 162)	0.692
Alanine aminotransferase (ALT) – IU/L‡	69 (17.9)	29 (15, 80)	29 (18, 67)	29 (15, 80)	0.579
Total bilirubin – mg/dL‡	71 (18.4)	0.7 (0.4, 1.9)	0.7 (0.4, 1.5)	0.7 (0.4, 2)	0.701
Direct bilirubin – mg/dL‡	72 (18.7)	0.3 (0.2, 1.4)	0.3 (0.2, 1.1)	0.3 (0.2, 1.5)	0.600
Indirect bilirubin – mg/dL‡	72 (18.7)	0.3 (0.2, 0.7)	0.3 (0.2, 0.6)	0.3 (0.2, 0.7)	0.719
Final diagnosis – n (%)∫	0 (0)				
Septic shock		48 (12.5)	12 (12.4)	36 (12.5)	1
Hyponatremia		47 (12.2)	6 (6.2)	41 (14.2)	0.047
Anemia		40 (10.4)	12 (12.4)	28 (9.7)	0.447
Hypertension		30 (7.8)	8 (8.3)	22 (7.6)	0.829
Atrial fibrillation (AF)		26 (6.8)	8 (8.3)	18 (6.3)	0.488
Sepsis		23 (6)	9 (9.3)	14 (4.9)	0.136
Acute kidney injury (AKI)		21 (5.5)	5 (5.2)	16 (5.6)	1
Cancer		16 (4.2)	4 (4.1)	12 (4.2)	1
Cirrhosis		12 (3.1)	3 (3.1)	9 (3.1)	1
Others		258 (67)	64 (66)	194 (67.4)	0.804
Treatment location when diagnosed as hyperkalemia – n (%)∫	0 (0)				0.045
Emergency department (ED)		178 (46.2)	47 (48.5)	131 (45.5)	0.639
Inpatient department (IPD)		159 (41.3)	32 (33)	127 (44.1)	0.057
Outpatient department (OPD)		48 (12.5)	18 (18.6)	30 (10.4)	0.049
In-hospital mortality – n (%)∫	0 (0)	82 (21.3)	27 (27.8)	55 (19.1)	0.275

*Abbreviations: BMI* body mass index, *g/dL* gram per deciliter, *IU* international unit, *kg/m*^*2*^ kilogram per square meter, *mg/dL* milligram per deciliter, *mm Hg* millimeter of mercury, *mm*^*3*^ cubic millimeter, *mmol/L* millimole per liter

^*^p < 0.05 was statistical significance

^†^Mean and standard deviation and the difference between groups were analyzed by *t*-test

^‡^Median (interquartile range) and the difference between groups were analyzed by Mann–Whitney U test

∫ Difference between groups were analyzed by Fisher’s exact test

^¶^According to a joint position statement of the American Diabetes Association and the European Association for the study of diabetes, level 1 hypoglycemia was a glucose level of  54-69 mg/dL (3.0-3.8 mmol/L). Level 2 hypoglycemia was blood glucose level < 54 mg/dL or < 3.0 mmol/L. Level 3 was severe hypoglycemia with severe cognitive impairment requiring external assistance for recovery (Diabetes Care. 2017 Jan;40(1):155–7 and Diabetes Care. 2022 Jan;45(Suppl 1):S83–96)

### Model development

After multivariable logistic regression modeling with backward elimination, there were three significant predictors in the reduced model as follows: age > 60 years old (β coefficient = 0.72, *p* 0.004), pretreatment blood glucose ≤ 100 mg/dL (≤ 5.6 mmol/L) (β coefficient = 0.88, *p* < 0.001), and pretreatment serum potassium > 6 mmol/L (β coefficient = 0.65, *p* 0.018).

### Score transformation

According to the multivariable reduced model, we divided the β coefficients for each predictor by 0.65 (the lowest β coefficient of all predictors) and multiplied by 2 to transform the score from decimal into integers. For age (> 60 years old) score was 2 points, the pretreatment blood glucose (≤ 100 mg/dL or ≤ 5.6 mmol/L) score was 3 points, and the pretreatment serum potassium (> 6 mmol/L) score was 2 points. The total assigned transformation score was 7 points (Table 2).

**Table 2:** Univariable, multivariable logistic regression model with full and reduced models to predict posttreatment hypoglycemia (blood glucose ≤ 70 mg/dL or ≤ 3.9 mmol/L)

Variables	Univariable			Multivariable (Full model) †			Multivariable (Reduced model) ‡			Assigned risk item score
	**Odds ratio (95% CI)**	*p*-value*	**AuROC (95% CI)**	**Odds ratio (95% CI)**	*p*-value*	β	**Odds ratio (95% CI)**	*p*-value*	β	
Age – year										
≤ 60	Ref			Ref			Ref			
> 60	1.83 (1.15—2.92)	0.011	0.575 (0.518—0.632)	2.06 (1.26—3.37)	0.004	0.72	2.06 (1.27—3.33)	0.004	0.72	2
Female	0.97 (0.60—1.55)	0.896	0.496 (0.440—0.553)	0.88 (0.53—1.45)	0.613	-0.13				
BMI – kg/m^2^										
< 18.5	1.44 (0.80—2.59)	0.223	0.527 (0.481—0.572)	1.42 (0.76—2.64)	0.272	0.35				
≥ 18.5	Ref			Ref						
Pretreatment blood glucose – mg/dL										
≤ 100 (≤ 5.6 mmol/L)	2.36 (1.47—3.79)	< 0.001	0.600 (0.544—0.656)	2.32 (1.42—3.79)	0.001	0.84	2.4 (1.48—3.9)	< 0.001	0.88	3
> 100 (> 5.6 mmol/L)	Ref			Ref			Ref			
Pretreatment serum potassium – mmol/L										
≤ 6	Ref			Ref			Ref			
> 6	1.85 (1.10—3.11)	0.020	0.566 (0.514—0.617)	1.97 (1.13—3.43)	0.017	0.68	1.91 (1.12—3.26)	0.018	0.65	2
Pretreatment serum creatinine – mg/dL										
≤ 3.3 (≤ 291.7 µmol/L)	Ref			Ref						
> 3.3 (> 291.7 µmol/L)	1.26 (0.79—2.00)	0.335	0.528 (0.471—0.586)	1.19 (0.73—1.94)	0.492	0.17				

*Abbreviations: AuROC* area under receiver operating characteristic curve, *BMI* body mass index, *kg/m*^*2*^ kilogram per square meter, *mg/dL* milligram per deciliter, *mmol/L* millimole per liter, *µmol/L* micromole per liter, *Ref* reference, *β* regression coefficient, *95% CI * 95% confidence interval

^*^ *p* < 0.05 was statistical significance

^†^The AuROC of full model was 0.662 (95% CI 0.597 to 0.726)

^‡^The AuROC of reduced model was 0.657 (95% CI 0.596 to 0.718)

To simplify the risk score transformation, we categorized the sum of scores as the levels of risk categories. We divided the risk categories into 3 options with a cut-off at 2, 3, and 4 points to separate low-risk and high-risk patients. We demonstrated our scoring system's sensitivity, specificity, PPV, NPV, LHR + , LHR-, and accuracy (Table 3).

**Table 3:** Distribution of posttreatment blood glucose ≤ 70 mg/dL (≤ 3.9 mmol/L) across different levels of risk categories

Risk categories^a^	Score	Posttreatment hypoglycemia – n (%) (*n* = 97)	No posttreatment hypoglycemia – n (%) (*n* = 288)	Sensitivity (95% CI)	Specificity (95% CI)	PPV (95% CI)	NPV (95% CI)	LHR + (95% CI)	LHR- (95% CI)	Accuracy (95% CI)
**Option 1**										
Low	< 2	4 (4.1)	43 (14.9)	95.9 (89.8—98.9)	14.9 (11—19.6)	27.5 (22.8—32.6)	91.5 (79.6—97.6)	1.13 (1.06—1.2)	0.28 (0.1—0.75)	35.3 (30.6—40.3)
High	≥ 2	93 (95.9)	245 (85.1)							
**Option 2**										
Low	< 3	30 (30.9)	152 (52.8)	69.1 (58.9—78.1)	52.8 (46.8—58.7)	33 (26.6—39.9)	83.5 (77.3—88.6)	1.46 (1.22—1.75)	0.59 (0.43—0.8)	56.9 (51.8—61.9)
High	≥ 3	67 (69.1)	136 (47.2)							
**Option 3**										
Low	< 4	34 (35.1)	165 (57.3)	64.9 (54.6—74.4)	57.3 (51.4—63.1)	33.9 (27.1—41.2)	82.9 (77—87.9)	1.52 (1.25—1.85)	0.61 (0.46—0.82)	59.2 (54.1—64.2)
High	≥ 4	63 (65)	123 (42.7)							

*Abbreviations: LHR +*  positive likelihood ratio, *LHR-* negative likelihood ratio, *NPV* negative predictive value, *PPV* positive predictive value, *95% CI* 95% confidence interval

^a^Using model: For age (> 60 years old) score was 2 points, pretreatment blood glucose (≤ 100 mg/dL or ≤ 5.6 mmol/L) score was 3 points, and pretreatment serum potassium (> 6 mmol/L) score was 2 points. The total assigned transformation score was 7 points

Using a cut-off point of 2 points to classify as high (score ≥ 2 points) and low-risk groups (score < 2 points) among patients with posttreatment hypoglycemia, the median (IQR) of the duration of posttreatment hypoglycemia in high and low-risk groups were 249 min (190 to 428 min) and 424 min (304 to 526 min), respectively.

Using a cut-off point of 3 points to classify as high (score ≥ 3 points) and low-risk groups (score < 3 points) among patients with posttreatment hypoglycemia, the median (IQR) of the duration of posttreatment hypoglycemia in high and low-risk groups were 251 min (190 to 435 min) and 260 min (211 to 424 min), respectively.

Using a cut-off point of 4 points to classify as high (score ≥ 4 points) and low-risk groups (score < 4 points) among patients with posttreatment hypoglycemia, the median (IQR) of the duration of posttreatment hypoglycemia in high and low-risk groups were 251 min (181 to 470 min) and 260 min (211 to 424 min), respectively.

### Model performances and internal validation

The AuROC of full model was 0.662 (95% CI 0.597 to 0.726). The AuROC of the reduced model was 0.657 (95% CI 0.596 to 0.718).

**Fig. 2:**
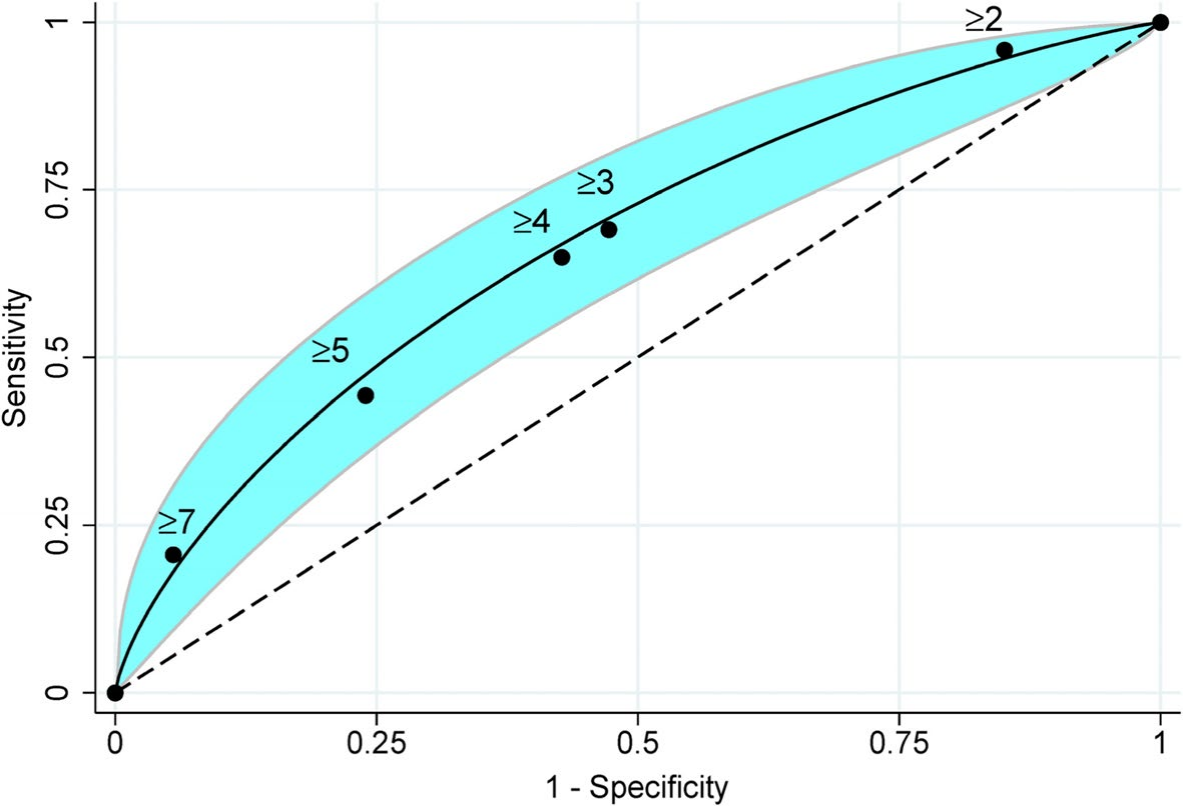
Discrimination plot of the predictive score. Parametric receiver operating characteristic curve (ROC) showed the area under ROC = 0.671 (95% confidence interval = 0.608 to 0.735); dots and numbers represented each cut-off points; the light blue band represented 95% confidence interval

**Fig. 3:**
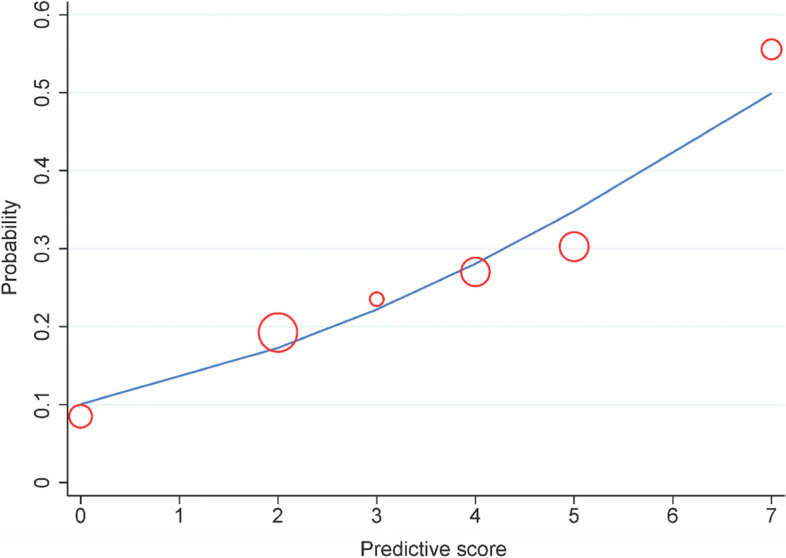
Calibration plot of the predictive score. Observed risk (red circle) versus predicted risk score (solid blue line) of posttreatment blood glucose ≤ 70 mg/dL (≤ 3.9 mmol/L). The more circles near the solid blue line indicated the more accuracy in predicting the risk score of posttreatment blood glucose, and this figure showed acceptable accuracy. The size of the circle represented the frequency of posttreatment blood glucose in each total score

After score transformation, parametric ROC showed the AuROC of 0.671 (95% CI 0.608 to 0.735) (Fig. 2). The calibration plot of the predictive score showed consistency with the original data (Fig. 3). Hosmer–Lemeshow goodness-of-fit test showed no evidence of lack-of-fit of the model (*p* 0.792); therefore, the model was fit to the original data.

Internal validation via bootstrap sampling showed a consistent AuROC of 0.670 (95% CI 0.660 to 0.670) with minimal model optimism at 0.014 (range -0.087 to 0.098).

**Fig. 4:**
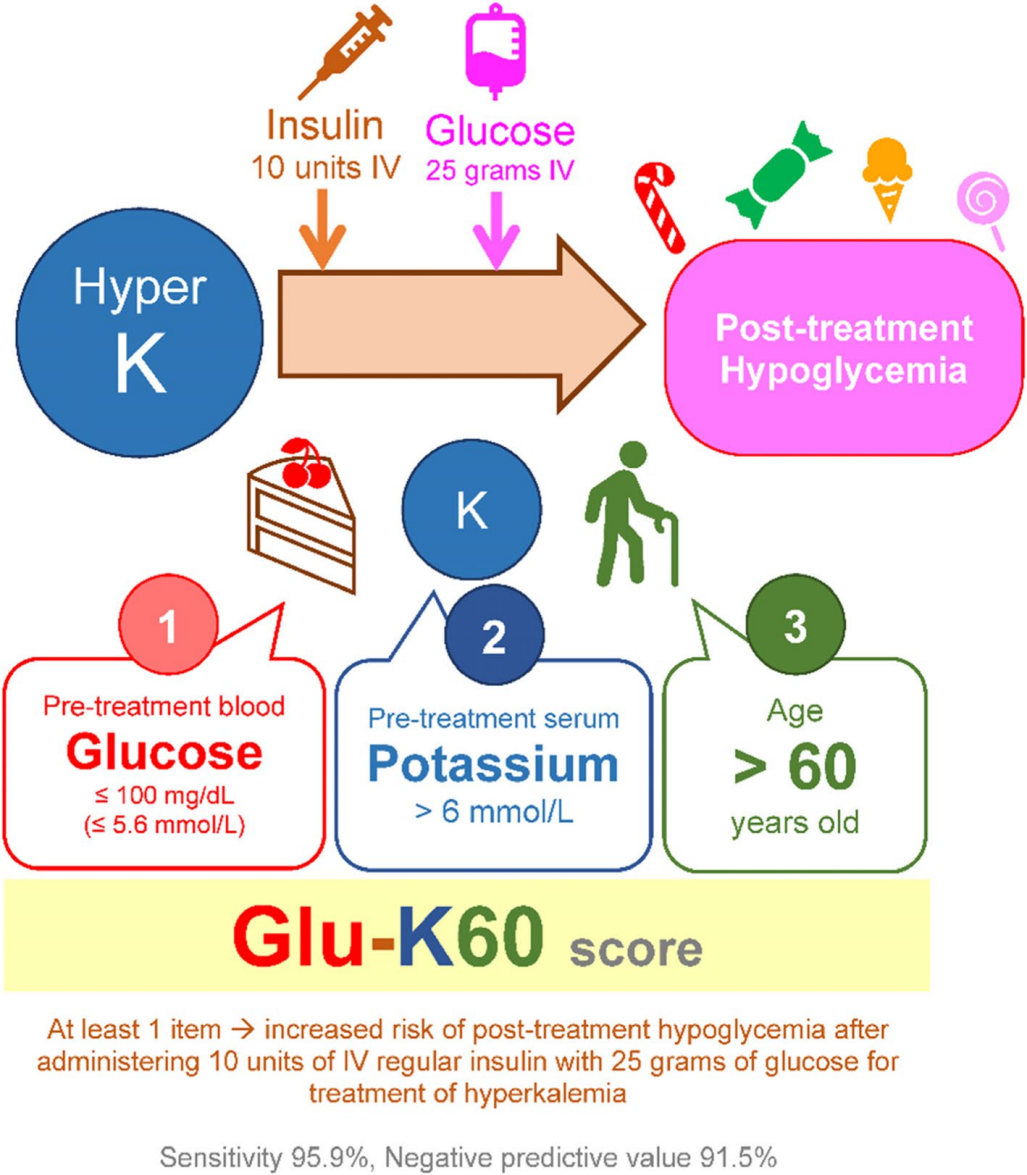
Illustration of predictive score

Our predictive score was named “Glu-K60 score” to represent the contributed predictors as pretreatment blood glucose (“Glu”), pretreatment serum potassium (“K”), and age > 60 years old (“60”) (Fig. 4).

### Post-hoc analysis using posttreatment blood glucose < 54 mg/dL (< 3.0 mmol/L) as posttreatment level 2 hypoglycemia and level 3 hypoglycemia

Level 2 hypoglycemia (blood glucose level < 54 mg/dL or < 3.0 mmol/L) and level 3 hypoglycemia (severe hypoglycemia regarding severe cognitive impairment requiring external assistance for recovery) were pooled as one group and analyzed as posttreatment hypoglycemia according to a joint position statement of the American Diabetes Association and the European Association for the study of diabetes [19, 27].

Multivariable logistic regression modeling with backward elimination was performed step-by-step to derive the full model and the reduced model. In the full model, age > 60 years old, pretreatment blood glucose ≤ 100 mg/dL (≤ 5.6 mmol/L), and pretreatment serum potassium > 6 mmol/L demonstrated statistical significance and were sent into the next round to be the reduced model round #1. In the reduced model round #1, age > 60 years old, pretreatment blood glucose ≤ 100 mg/dL (≤ 5.6 mmol/L) was statistically significant (the AuROC: 0.638, 95% CI 0.566 to 0.710) and sent into the reduced model round #2. In the reduced model round #2, pretreatment blood glucose ≤ 100 mg/dL (≤ 5.6 mmol/L) was statistically significant (the AuROC: 0.631, 95% CI 0.553 to 0.710) and sent into the reduced model round #3. In the reduced model round #3, a pretreatment blood glucose ≤ 100 mg/dL (≤ 5.6 mmol/L) was still statistically significant (the AuROC: 0.595, 95% CI 0.522 to 0.668). However, there was a borderline significant decrease in the AuROC between round #2 and round #3 (*p*-value of likelihood-ratio test of 0.065). Moreover, aging can alter glucose counter-regulation by various mechanisms in a previous study [28]. Age was regarded as a clinically significant variable rather than a statistically significant variable. Thus, the reduced model round #2 (where included age in the model) was used as the final model to assign item scores to optimize the AuROC (Table 4).

**Table 4:** Univariable, multivariable logistic regression model with full and reduced models to predict posttreatment level 2 hypoglycemia (blood glucose < 54 mg/dL or < 3.0 mmol/L) and level 3 hypoglycemia

Variables	Univariable			Multivariable (Full model) †		Multivariable (Reduced model round #1) ‡		Multivariable (Reduced model round #2) ∫			Reduced model round #3 ¶		Assigned risk item score from reduced model round #2
	**Odds ratio (95% CI)**	*p*-value*	**AuROC (95% CI)**	**Odds ratio (95% CI)**	*p*-value*	**Odds ratio (95% CI)**	*p*-value*	**Odds ratio (95% CI)**	*p*-value*	β	**Odds ratio (95% CI)**	*p*-value*	
Age – year													
≤ 60	Ref			Ref		Ref		Ref					
> 60	1.68 (0.93—3.04)	0.084	0.565 (0.492—0.638)	1.89 (1.02—3.50)	0.043	1.85 (1.01—3.39)	0.045	1.75 (0.96—3.19)	0.067	0.56			2
Female	0.67 (0.36—1.25)	0.209	0.546 (0.477—0.615)	0.58 (0.30—1.12)	0.102								
BMI – kg/m^2^													
< 18.5	1.84 (0.92—3.69)	0.085	0.548 (0.486—0.611)	1.94 (0.93—4.04)	0.077								
≥ 18.5	Ref			Ref									
Pretreatment blood glucose – mg/dL													
≤ 100 (≤ 5.6 mmol/L)	2.23 (1.24—4.03)	0.008	0.595 (0.522—0.668)	2.04 (1.11—3.75)	0.021	2.23 (1.22—4.06)	0.009	2.30 (1.27—4.16)	0.006	0.83	2.23 (1.24—4.03)	0.008	3
> 100 (> 5.6 mmol/L)	Ref			Ref		Ref		Ref			Ref		
Pretreatment serum potassium – mmol/L													
≤ 6	Ref			Ref		Ref							
> 6	1.90 (0.96—3.77)	0.065	0.566 (0.503—0.630)	2.26 (1.09—4.65)	0.028	1.93 (0.96—3.86)	0.065						
Pretreatment serum creatinine – mg/dL													
≤ 3.3 (≤ 291.7 µmol/L)	Ref			Ref									
> 3.3 (> 291.7 µmol/L)	1.11 (0.62—2.00)	0.725	0.513 (0.440—0.586)	1.06 (0.57—1.96)	0.855								

Abbreviations: AuROC, area under receiver operating characteristic curve; BMI, body mass index; kg/m^2^, kilogram per square meter; mg/dL, milligram per deciliter; mmol/L, millimole per liter; µmol/L, micromole per liter; Ref, reference; β, regression coefficient; 95% CI, 95% confidence interval

^*^ *p* < 0.05 was statistical significance. Reduced model was performed by using multivariable logistic regression modeling with backward elimination

^†^The AuROC of full model was 0.654 (95% CI 0.577 to 0.731)

^‡^The AuROC of reduced model round #1 was 0.638 (95% CI 0.566 to 0.710)

∫The AuROC of reduced model round #2 was 0.631 (95% CI 0.553 to 0.710). There was a decrease in the AuROC between round #2 and round #3. Thus, reduced model round #2 was used as the final model to assign item scores

^¶^The AuROC of reduced model round #3 was 0.595 (95% CI 0.522 to 0.668)

In the reduced model round #2, the two predictors were age > 60 years old (β coefficient = 0.56) and pretreatment blood glucose ≤ 100 mg/dL (≤ 5.6 mmol/L) (β coefficient = 0.83). We divided the β coefficients for each predictor by 0.56 (the lowest β coefficient of all predictors) and multiplied by 2 to transform the score from decimal into integers. For age (> 60 years old) score was 2 points and the pretreatment blood glucose (≤ 100 mg/dL or ≤ 5.6 mmol/L) score was 3 points. The total assigned transformation score was 5 points (Table 4). After score transformation, parametric ROC showed the AuROC of 0.644 (95% CI 0.560 to 0.728). Hosmer–Lemeshow goodness-of-fit test showed no evidence of lack-of-fit of the model (*p* 0.974); therefore, the model was fit to the original data.

To simplify the risk score transformation, we categorized the sum of scores as the levels of risk categories. We divided the risk categories into 2 options with a cut-off at 2 and 3 points to separate low-risk and high-risk patients. We demonstrated our scoring system's sensitivity, specificity, PPV, NPV, LHR + , LHR-, and accuracy (Table 5).

**Table 5:** Distribution of posttreatment level 2 hypoglycemia (blood glucose < 54 mg/dL or < 3.0 mmol/L) and level 3 hypoglycemia across different levels of risk categories

Risk categories^a^	Score	Posttreatment hypoglycemia – n (%) (n = 97)	No posttreatment hypoglycemia – n (%) (*n* = 288)	Sensitivity (95% CI)	Specificity (95% CI)	PPV (95% CI)	NPV (95% CI)	LHR + (95% CI)	LHR- (95% CI)	Accuracy (95% CI)
**Option 1**										
Low	< 2	10 (19.2)	124 (37.2)	80.8 (67.5—90.4)	37.2 (32—42.7)	16.7 (12.3—21.9)	92.5 (86.7—96.4)	1.29 (1.1—1.5)	0.52 (0.29—0.92)	43.1 (38.1—48.2)
High	≥ 2	42 (80.8)	209 (62.8)							
**Option 2**										
Low	< 3	26 (50)	230 (69.1)	50 (35.8—64.2)	69.1 (63.8—74)	20.2 (13.6—28.1)	89.8 (85.5—93.3)	1.62 (1.18—2.22)	0.72 (0.55—0.96)	66.5 (61.5—71.2)
High	≥ 3	26 (50)	103 (30.9)							

*Abbreviations: LHR +*  positive likelihood ratio, *LHR-* negative likelihood ratio, *NPV* negative predictive value, *PPV* positive predictive value, *95% CI* 95% confidence interval

^a^Using model: For age (> 60 years old) score was 2 points and pretreatment blood glucose (≤ 100 mg/dL or ≤ 5.6 mmol/L) score was 3 points. The total assigned transformation score was 5 points

Internal validation via bootstrap sampling showed a consistent AuROC of 0.638 (95% CI 0.636 to 0.640) with minimal model optimism at 0.014 (range -0.101 to 0.198).

## Discussion

### Significance of main findings

This study used a retrospective observational cohort of hyperkalemic patients treated with 10 units of IV regular insulin and 25 g of glucose. We developed the predictive score comprising age > 60 years old, pretreatment blood glucose ≤ 100 mg/dL (≤ 5.6 mmol/L), and pretreatment potassium > 6 mmol/L to predict posttreatment hypoglycemia complicating from insulin treatment in the hyperkalemic patient. Its predictive performance was fair discrimination.

Hypoglycemia after treatment of hyperkalemia ranges from 8.7% to 75% [6, 9, 11–13]. Similarly, our study found an incidence of posttreatment hypoglycemia was 25.2% among patients in the ED, OPD, and IPD. Of these, patients in OPD had an increased risk of posttreatment hypoglycemia compared to IPD. Those results might be due to a lack of close monitoring of hypoglycemic symptoms and delayed recognition of hypoglycemia. So, delayed recognition by medical personnel led to the patient's blood glucose turning low until the patient experienced hypoglycemic symptoms and signs. Eventually, medical personnel began to check the blood glucose and showed hypoglycemia.

Signs and symptoms of hypoglycemia include palpitation, tachycardia, diaphoresis, tremor, anxiety, hunger, shakiness, irritability, confusion, altered mental status, seizure, and death [27, 29, 30]. We found the median (IQR) of the duration of hypoglycemia was 253 min (190 to 435 min) among patients with posttreatment hypoglycemia. Therefore, observation of posttreatment hypoglycemia should cover 3 to 7 h. Based on the median duration of posttreatment hypoglycemia in every cut-off point of our score among patients with posttreatment hypoglycemia, we found high risk and low-risk groups experienced hypoglycemia ranging from 249 to 251 min (approximately 4.5 h) and 260 to 424 min (approximately 7 h), respectively. Similarly, Tee et al. demonstrated that most hypoglycemia occurred within 3 h of treatment [12]. Meanwhile, Wheeler et al. reported that most hyperglycemic patients occurred within the first 6 h [18]. According to our results, we suggested a regular re-evaluation of hypoglycemic manifestations or checking blood glucose in high risk. For example, in high-risk, we proposed re-evaluating the hypoglycemic symptoms every 1 to 2 h and checking blood glucose when the patient had hypoglycemic symptoms until 4.5 h, depending on the types of insulin. In low risk, we proposed re-evaluating the hypoglycemic symptoms every 3 to 4 h and checking blood glucose when the patient had hypoglycemic signs or symptoms until 7 h.

Intravenous glucose administration could stimulate endogenous insulin secretion; however, previous pieces of literature did not recommended using intravenous glucose without exogenous insulin administration. Patients with insulin-dependent diabetes or inadequate insulin reserve might have inadequate endogenous insulin secretion. Those patients experienced hyperglycemia resulting in increased plasma osmolarity, then enhanced intracellular potassium shifted into the extracellular fluid compartment and worsened hyperkalemia [16, 31]. That was a limitation of most previous studies, which included the diabetic patient, influencing the results [11, 12, 16, 26]. Moreover, previous studies reported that patients with no prior diagnosis of diabetes had an increased risk of posttreatment hypoglycemia [11, 16]. Focusing on non-diabetic patients might be a benefit for predicting posttreatment hypoglycemia. Therefore, the strength of our study was the excluded diabetic patients and represented the non-diabetic population.

Aging can alter the glucose counter-regulation by dysregulating glucagon, growth hormone, and epinephrine responses to hypoglycemia and putting the elderly patient at risk of having hypoglycemia [28]. Our predictive score showed that an elderly patient was likely to have posttreatment hypoglycemia. Although the fasting plasma glucose levels increased with age at a rate of 0.7 to 1.1 mg/dL (0.039 to 0.061 mmol/L) per age decade among non-diabetic patients, the basal hepatic glucose production was lower at older ages [22]. However, Crnobrnja et al. and Apel et al.’s studies showed no association between age and posttreatment hypoglycemia [11, 16]. Further investigation might be conducted to explore this association.

In an animal study, estrogen (estradiol or E2) acts via the estrogen receptor α-phosphoinositide 3-kinase (PI3K)-Akt-Foxo1 signaling resulting in increased glucose uptake leading to females tend to develop hypoglycemia due to the influence of estrogen [32]. In a human study, Wheeler et al. reported that females were associated with an increased risk of hypoglycemia [18]. However, our result and Tee et al. demonstrated that sex was not associated with posttreatment hypoglycemia. The existing difference in those results might be from a difference in intravenous insulin dosing. Wheeler et al. compared weight-based insulin dosing (0.1 units/kg of body weight up to a maximum of 10 units) and standard fixed dosing (10 units) in a patient with weight less than 95 kg and measured the endpoint as blood glucose < 70 mg/dL (< 3.9 mmol/L) within 24 h. Females in the standard fixed dosing group (10 units of insulin) were associated with an increased risk of hypoglycemia (OR 3.2, 95%CI 1.1 to 9.1, *p* 0.03) compared to the weight-based insulin dosing group (≤ 10 units of insulin) [18]. We hypothesized that the formulae 0.1 units/kg of body weight would result in lower total insulin doses in females (who had generally lower body weight than males) compared to fixed doses of 10 units. It could lead to a lower incidence of hypoglycemia in females than males in the weight-based insulin dosing group. Eventually, the incidence of hypoglycemia in the standard fixed dosing group of females compared to males was more than the incidence of hypoglycemia in the weight-based insulin dosing group of females compared to males and increased the risk of hypoglycemia in females, as they reported. Meanwhile, all our patients and patients in Tee et al.’s study were given fixed dose of 10 units of regular insulin [12].

Patients with lower BMI tend to have less hepatic glycogen storage, decreased glucagon and epinephrine secretion, and poor glycogenolysis and gluconeogenesis [23]. Crnobrnja et al. reported that higher BMI was a protective factor of posttreatment hypoglycemia (OR 0.83, 95% CI 0.69 to 0.99, *p* 0.048) [11]. However, Apel et al.’s and our study demonstrated no evidence of a difference between BMI groups. BMI measurement was less obtained in our setting, including weight and height (our missing data of BMI, weight, and height was 22.3%, 19.7%, and 22.3%, respectively). The data imputation procedure might not be better than using actual data, leaving could not be demonstrated the difference. On the other hand, the benefit of BMI as a non-contributing factor in the reduced model of our predictive score was more practical for the ED or any emergency conditions in which BMI might be inconvenient to obtain. When weight, height, and BMI are required before initiating treatment, a treatment delay has possibly occurred.

Our study found that low pretreatment blood glucose increased the risk of posttreatment hypoglycemia. Similarly, Tee et al. reported that pretreatment blood glucose between patients with and without posttreatment hypoglycemia were 106.2 mg/dL (5.9 mmol/L) and 136.8 mg/dL (7.6 mmol/L) (*p* < 0.001), respectively [12]. Also, Apel et al. revealed that pretreatment blood glucose between patients with and without posttreatment hypoglycemia were 104 mg/dL (5.8 mmol/L) and 162 mg/dL (9 mmol/L) (*p* 0.04), respectively [16]. In addition, Crnobrnja et al. found that higher pretreatment blood glucose was a protective factor against posttreatment hypoglycemia (OR 0.88, 95% CI 0.81 to 0.95, *p* 0.002) [11].

Our study showed that high pretreatment serum potassium increased the risk of posttreatment hypoglycemia. In contrast, Tee et al. found that less pretreatment serum potassium increased the risk of posttreatment hypoglycemia (median potassium 6.1 mmol/L in the hypoglycemic group and 6.3 mmol/L in the no hypoglycemic group, *p* 0.024) [12]. The reason for this issue was a difference in eligibility criteria for hyperkalemic levels and diabetic patients. Tee et al. included the patients with pretreatment serum potassium ≥ 6.5 mmol/L or 6 to 6.4 mmol/L with electrocardiography change and previous diagnosis of diabetes. While the inclusion criterion of our study was pretreatment serum potassium > 5.3 mmol/L, and we excluded the diabetic patients. The wider range of pretreatment serum potassium in our study could be more generalizable in real-world practice.

Previous studies proposed that patients with chronic kidney disease (CKD) and end-stage kidney disease (ESKD) were susceptible to hypoglycemia due to reduced insulin clearance in the kidney; declined renal and hepatic glucose production; decreased gluconeogenesis during uremia; increased glucose uptake of red blood cell during hemodialysis; impaired counterregulatory hormone responses such as cortisol, and growth hormone; and nutritional deprivation [33–36]. Garcia et al. found a higher baseline serum creatinine was related to a high incidence of posttreatment hypoglycemia (OR 1.12, 95% CI 1.02 to 1.23, *p* 0.016) [26]. However, Tee et al. reported that serum creatinine was not related to posttreatment hypoglycemia (median creatinine 2.78 mg/dL [246 µmol/L] in the hypoglycemic group and 1.92 mg/dL [170 µmol/L] in the non-hypoglycemic group, *p* 0.669) [12]. Similarly, our study found no association between pretreatment serum creatinine and hypoglycemia. Most of our patients had impaired renal function, which was demonstrated with preexisting high pretreatment serum creatinine (median 3.7 mg/dL [327 µmol/L], IQR 1.8 to 8.5 mg/dL [159 to 752 µmol/L]) and ESKD (29.6%) and CKD (12%) as comorbidity. Thus, the effect of impaired renal function did not contribute to the posttreatment hypoglycemic prediction in our model.

Strategies were aimed to prevent posttreatment hypoglycemia with preserved efficacy of lowering serum potassium. Wheeler et al. investigated using a retrospective chart review to compare the effect of weight-based insulin dosing (0.1 units/kg of body weight up to a maximum of 10 units or weight less than 95 kg), and standard fixed dosing (10 units regardless of body weight) with both groups receiving 50 g of dextrose for treatment of hyperkalemia and measuring endpoint as blood glucose < 70 mg/dL (< 3.9 mmol/L) within 24 h after insulin administration. Weight-based insulin dosing reduced hypoglycemic events compared to standard fixed dosing (12.1% versus 27.3%, *p* 0.05), which preserved potassium lowering effects (average potassium decrease 1.34 mmol/L versus 1.35 mmol/L, *p* 0.94) [18]. Garcia et al. reported using a retrospective cohort study to compare 5 units and 10 units of IV insulin. The results showed that using 5 units of IV insulin reduced the risk of posttreatment hypoglycemia compared to 10 units of IV insulin (OR 0.307, 95% CI 0.117 to 0.806), which preserved potassium lowering effects (potassium difference -0.096 mmol/L, 95%CI -0.250 to 0.058, *p* 0.221) [26].

However, a strategy that could not prevent posttreatment hypoglycemia as follows. Farina et al. reported using a multicenter, retrospective, matched cohort study to compare administering 25 g and 50 g of glucose in addition to 10 units of IV insulin to treat hyperkalemia. The result showed no difference in the incidence of hypoglycemia between the 25 g of glucose group and 50 g of glucose group (15.8% compared to 8.3% of patients who developed hypoglycemia, *p* 0.11) at 60 min following the treatment. Hyperglycemia occurred at 60 min posttreatment among patients who received the 50 g of glucose but did not remain at 240 min. Meanwhile, no difference was seen in potassium reduction at 60 min across groups [37].

We simplified the predictive score and categorized the sum of scores as levels of risk categories to predict the posttreatment blood glucose ≤ 70 mg/dL (≤ 3.9 mmol/L). Three options with a cut-off at 2, 3, and 4 points. Our predictive score objective was used as a screening tool, so high sensitivity is paramount. A total score of ≥ 2 points (the highest sensitivity option) or met criteria at least one of the following: age > 60 years old, pretreatment blood glucose ≤ 100 mg/dL (≤ 5.6 mmol/L), or pretreatment serum potassium > 6 mmol/L indicated a high risk for posttreatment hypoglycemia in hyperkalemic patients treated with IV insulin and glucose. It might guide using weight-based insulin dosage (0.1 units/kg of body weight) [18], decreasing the IV insulin dosage from 10 to 5 units [26], or closely monitoring and frequently checking blood glucose levels in the high-risk group. A further prospective study or external validation study might be conducted to demonstrate the usefulness of our predictive score. However, our predictive score might be unsuitable to be a confirmation tool because of its low specificity.

According to a joint position statement of the American Diabetes Association and the European Association for the study of diabetes [19, 27], level 2 hypoglycemia (blood glucose level < 54 mg/dL or < 3.0 mmol/L) and level 3 hypoglycemia (severe hypoglycemia regarding severe cognitive impairment requiring external assistance for recovery) were pooled as one group and analyzed as posttreatment hypoglycemia in post-hoc analysis (Tables 4 and 5). We simplified the predictive score and categorized the sum of scores as levels of risk categories to predict posttreatment levels 2 and 3 hypoglycemia. Two options with a cut-off at 2 and 3 points. To use this predictive score as a screening tool, so high sensitivity is essential. A total score of ≥ 2 points (the highest sensitivity option) or met criteria at least one of the following: age > 60 years old or pretreatment blood glucose ≤ 100 mg/dL (≤ 5.6 mmol/L) indicated a high risk for posttreatment levels 2 and 3 hypoglycemia in hyperkalemic patients treated with IV insulin and glucose. For early detection purpose of posttreatment hypoglycemia, the level 1 hypoglycemia should be used for early treatment before the patient moved into level 2 hypoglycemia (less than 54 mg/dL or 3.0 mmol/L) or level 3 hypoglycemia. In addition, level 2 and level 3 hypoglycemia were pooled as one group and analyzed as posttreatment hypoglycemia without separating level 2 and level 3 hypoglycemic patients into two groups to prevent statistical bias from a multiple testing problem in post-hoc analysis. Classification of the posttreatment hypoglycemia into levels 1, 2, and 3 hypoglycemia might be used as an endpoint in a prespecified method in research protocols in further research.

### Limitations

This study had some limitations. First, the retrospective observational cohort design made it difficult to control other factors influencing the results. The quality of data depended on the available historical data. Some data were unavailable in the medical records of some patients, such as weight, height, BMI, and pretreatment blood glucose. Especially patients who visited the ED or had any conditions where the data of weight, height, BMI, and pretreatment blood glucose were not routinely measured because it may delay the treatment such as life-threatening conditions or cardiac arrest. So, we prespecified the plan in our study protocol to use the most recent previous data within three months if the weight, height, or BMI data was unavailable during the hyperkalemic visit. However, it might not be up-to-date actual data. Therefore, a further prospective design could improve this issue.

Second, the AuROC of the predictive score was 0.671 (95% CI 0.608 to 0.735), which showed fair discrimination. Other predictors should be investigated in the further study; it might increase the predictive performance. However, the number of predictors and the performance outcome should be balanced for practicality: the more required predictors, the more data needed to be completely obtained and the more challenging its use. There was a burden in settings with limited resources or time constraints like the ED. Our strength was that our predictive score required only three essential predictors led to easier use in real-world practice.

Third, acute kidney injury can cause hyperkalemia. Because of the retrospective design, there was no accurately available data on the cause of hyperkalemia for each patient provided by the attending physician. In a further study, possible causes of hyperkalemia in each patient should be collected in a prospective design.

Fourth, a repeated dose of the insulin or glucose can affect the posttreatment blood glucose level. Although there was no prespecified data collection regarding who received the repeated dose of the treatment, there were planned to use the lowest posttreatment blood glucose level within 12 h after the first insulin administration for analysis of posttreatment blood glucose level. If the patient had a repeated dose of insulin or glucose (during the period of assessment the endpoint as the posttreatment hypoglycemia), only the lowest blood glucose before being given the repeated dose would be determined as the posttreatment blood glucose level.

Fifth, this study assessed glucose levels by point-of-care testing for glucose (glucose POCT). Although a previous study reported a slight difference between glucose levels from glucose POCT and central laboratory testing [38], glucose POCT would be convenient for continuous glucose monitoring and increase the early detection of hypoglycemic incidence. In addition, the glucose POCT result was acceptably close to the central laboratory result if blood glucose < 100 mg/dL (< 5.6 mmol/L), as in patients at risk for hypoglycemia [38].

Sixth, the patient in this study was searched by using the ICD-10 code as hyperkalemia code in our hospital electronic medical records, which could not begin with searching the potassium level in the pooled data of laboratory results and then linked to the patient for retrieving the list of the eligible patients. This was a limitation of the hospital in a developing country with incomplete function of hospital electronic medical records or available only in a paper-based system. Retrieving the patient diagnosed with hyperkalemia solely from the ICD-10 code might include the patient with relatively severe hyperkalemia and encoded as hyperkalemia in the final diagnosis in ICD-10. In the research method design in the further study, the eligible patient might be searched from potassium level in the laboratory results and ICD-10 code in the hospital electronic medical record if the system is available.

## Conclusions

A total score ≥ 2 points or met criteria at least one of the following: age > 60 years old, pretreatment blood glucose ≤ 100 mg/dL (≤ 5.6 mmol/L), or pretreatment potassium > 6 mmol/L indicated a high risk for posttreatment hypoglycemia in hyperkalemic patients treated with IV insulin and glucose. Frequently checking blood glucose levels should be performed in the high-risk group.

## Data Availability

The datasets generated and/or analyzed during the current study are not publicly available due to privacy restrictions but are available from the corresponding author upon reasonable request.

## Abbreviations

AF: Atrial fibrillation
AKI: Acute kidney injury
ALP: Alkaline phosphatase
ALT: Alanine aminotransferase
AST: Aspartate aminotransferase
AuROC: Area under the receiver operating characteristic curve
β coefficient: Regression coefficient
BMI: Body mass index
BUN: Blood urea nitrogen
CKD: Chronic kidney disease
ED: Emergency department
ESKD: End-stage kidney disease
Glucose POCT: Point-of-care testing for glucose
g/dL: Gram per deciliter
ICD-10: 10^th^ revision of the International Statistical Classification of Diseases and Related Health Problems
IPD: Inpatient department
IQR: Interquartile range
IU: International unit
IV: Intravenous
kg/m^2^: Kilogram per square meter
LHR +: Positive likelihood ratio
LHR-: Negative likelihood ratio
MAR: Missing at random
MCAR: Missing completely at random
mg/dL: Milligram per deciliter
mm^3^: Cubic millimeter
mm Hg: Millimeter of mercury
mmol/L: Millimole per liter
Na^+^-K^+^ ATPase: Sodium–potassium-adenosine triphosphatase
NHE1: Na + -H + antiporters (sodium/hydrogen exchanger-1)
NPV: Negative predictive value
OPD: Outpatient department
OR: Odds ratio
PI3K: Phosphoinositide 3-kinase
PPV: Positive predictive value
REDCap: Research Electronic Data Capture
Ref: Reference
ROC: Receiver operating characteristic curve
SpO_2_: Oxygen saturation
TRIPOD: Transparent reporting of a multivariable prediction model for individual prognosis or diagnosis Statement
µmol/L: Micromole per liter
95% CI: 95% Confidence interval
